# Bone metastasis as the first sign of gastric cancer

**DOI:** 10.11604/pamj.2017.28.95.13787

**Published:** 2017-09-29

**Authors:** Wafa Ben Ameur, Safa Belghali, Imen Akkari, Houneida Zaghouani, Elyes Bouajina, Elhem Ben Jazia

**Affiliations:** 1 Department of Internal Medicine and Infectious Diseases Farhat Hached Hospital Sousse, Tunisia; 2 Department of Rheumatology Farhat Hached Hospital Sousse, Tunisia; 3 Department of Radiology Farhat Hached Hospital Sousse, Tunisia

**Keywords:** Bone metastasis, gastric cancer, adenocarcinoma

## Abstract

The skeleton is a common metastatic site for visceral carcinomas. However, the presentation of gastric cancer as bony metastases without preceding gastrointestinal symptoms is rare which has been infrequently reported in the literature. We report an infrequent case of a 60-year-old patient diagnosed having a gastric carcinoma with bone metastasis as the first evidence. She has consulted with worsening backache which started two months priorly.

## Introduction

Bone is a common site of metastasis of carcinoma of prostrate, breast, lung, kidney, bladder and thyroid. Gastric cancer infrequently metastasizes to the bone. Disseminated bony metastases as the first clinical manifestation are seen in exceptional cases and it is known to have a very poor prognosis. The presentation of gastric cancer as bony metastases without preceding gastrointestinal symptoms is rare which has been infrequently reported in the literature. Here, we report a case of gastric carcinoma presenting with symptomatic bone metastases as first presentation. On examination, the patient had back tenderness without incontinence or walking disorders. However, reflexes were increased with positive Babinski at the left side. Biologic investigations showed an inflammatory syndrome, a high level of alkaline phosphatase (ALP) and cholestasis. Morphologic examinations revealed osteolytic lesions in the spine and pelvic mainly. The research of the primary found a small gastric lesion. A biopsy indicated an adenocarcinoma moderately differentiated and infiltrating of the stomach. The patient underwent chemotherapy with oxaliplatin and 5-fluorouracil along with radiation.

## Patient and observation

**Figure 1:**
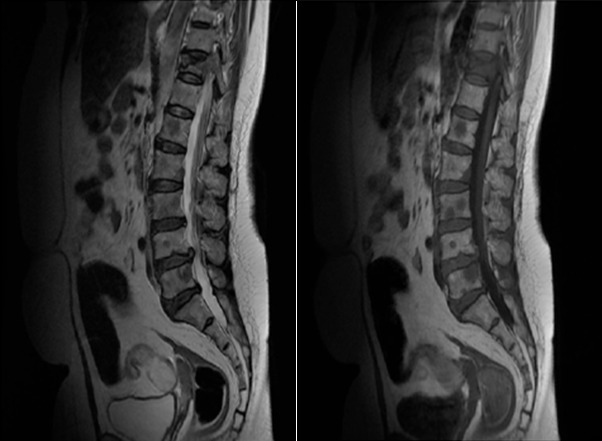
Radiological aspects of bone metastases on MRI images in T1 and T2 sequences

We present a rare case of a middle aged female with a history of rheumatoid arthritis under non-steroidal anti-inflammatory drugs which is currently in remission. Since 2 months, she describes inflammatory lower back pain without a preceding trauma. She denied nausea, vomiting, hematochezia, melena or weight loss. His family history was negative for any malignancies. On examination the patient had fever 39°C, pain in the pressure of the thorny of dorsal and lumbar vertebrae. She had normal strength and sensation in the lower extremities and denied any fecal or urinary incontinence. However, reflexes were increased with positive Babinski at the left side. Investigations showed Hb of 7, 2 g/dl, WBC count 8600/mm^3^, hyper eosinophilia of 1100/mm^3^, platelets 80,000/mm^3^, sedimentation rate 135/mm^3^, C-reactive protein 81 mg/l, alkaline phosphatase (ALP) 466 U/l (reference range 35-140 IU/L), Calcium and prothrombin time 80% of normal. Renal function was normal. We noted a cholestasis (2 x ULN) with a normal rate of bilirubine. X ray of the spine revealed several lytic lesions throughout most of his spine which initially raised suspicion of metastasis. Magnetic resonance imaging showed secondary lesions in the spine and pelvis. Bone scintigraphy revealed abnormal uptake in the spine (T5, T9 and T11), ribs and in sacro iliac joint. The patient was then investigated to look for the primary. Echo mammography was normal. CT scan of abdomen and pelvis showed multiples hepatic metastasis. Uppergasrointestinal endoscopy was done and revealed a small 2 cm ulcerated mass at the antrum which was not visualized on the CT scan. A biopsy indicated an adenocarcinoma moderately differentiated and infiltrating of the stomach. The patient underwent chemotherapy with oxaliplatin and 5-fluorouracil along with radiation Figure 1.

## Discussion

Gastric cancer is still the second leading cause of cancer-related deaths worldwide [1]. It generally metastasizes to the peritoneal membrane, liver, lymph nodes, etc and it may metastasize to the spleen, adrenalin, ovary, lung, brain and skin. Bone metastasis in gastric cancer patients has been shown to be very rare and portend a poor prognosis [2]. In fact mean survival time is 4-5 months with bone metastases from gastric cancer [3]. In addition, Gastric cancer presenting as bone metastases without any preceding gastrointestinal symptoms has been infrequently reported in the literature [4]. The incidence of bone metastasis varies greatly among studies. In 1983, Yoshikawa and Kitaoka [5] reported that the incidence of bone metastasis is 1~20%. In 1987, Nishidoi and Koga [6] have reported that in 246 gastric cancer patients, bone metastasis was found in 13.4% of patients. Jae Bong Ahn et al [7] reported that among the 2,150 patients diagnosed with gastric cancer from June 1992 to August 2010, bone metastasis was associated in 19 patients for a frequency of 0.9%. The incidence of bones metastases varies widely from as low as 1% in clinical practice to as high as 45% in screening studies for bone metastases, implying that many cases are asymptomatic [8]. Bone metastasis may occur more frequently in cases with primary cancer with diffuse involvement of the stomach or a Borrmann type 4 morphology, poorly differentiated adenocarcinoma [9], signet ring cell carcinoma, which included a relatively younger age [10] and in cases with abundant lymph node metastasis in the vicinity. In bone metastasis, the cancer cells diffusely proliferate in the bone marrow and this can cause disseminated carcinomatosis; they also proliferate rapidly and thus induce bone destruction as well as hematological complications (disseminated vascular coagulation, hemolytic anemia and other hematological complications) [11]. The exact mechanism of spread of tumor cells to the bone is not known. Lenhert et al [12] proposed that the rich supply of blood capillaries in the gastric mucosa contributes to the early spread of cancer to the bone.

It is also suggested that an alternate non- portal route through the vertebral venous plexus may be the route of bone metastasis from gastric carcinoma. Bone scintigraphy could detect bone metastasis at the time approximately 3 months earlier than that with using plain X-rays [13]. But, hot uptake lesions may be detected in many others lesions such as Paget's disease and other metabolic bone diseases, degenerative arthritis, fractures, infectious bone diseases and other benign bone diseases or primary bone tumors and so it has limitations of low specificity. The accuracy of the diagnosis may be increased more by performing additional positron emission tomography-computed tomography, magnetic resonance imaging, bone marrow tapping or bone marrow histological tests in parallel. Several studies have reported that the vertebrae are the site where bone metastasis occurs most frequently [14]. These findings support that the vertebral vein system is the major route of bone metastasis. Choi et al [14] have reported that the most frequent sites of metastasis were the vertebrae (66%), the costa (59%), the pelvic bone (43%), the femur (30%) and the scapula and clavicle (17%). The most common clinical symptoms and complications of bone metastasis are bone pain, pathologic fracture and spinal cord compression. Various laboratory abnormalities that suggest the possibility of bone metastases include elevated alkaline phosphatase (ALP), increased LDH and anemia or thrombocytopenia. Yoshikawa and Kitaoka [15] have reported that in 23 gastric cancer patients associated with bone metastasis, radiation therapy was effective for the amelioration of bone pain. Of the same, McQuay et al [16] have reported that, 1 month after radiation therapy, the bone pain was resolved completely in 1/4 of the patients and at least 50% pain amelioration could be obtained in 1/3 of the patients. Chemotherapy for the treatment of advanced metastatic gastric carcinomas may be associated with better quality of life than symptomatic treatment alone. As described in previous studies, most patients with initial BM showed rapid and dramatic improvement in ECOG PS, haematologic abnormalities and ALP levels after chemotherapy [17]. The survival period of was recently improved by treatment with MTX+5FU, S1+paclitaxel, S1+cisplatin and other chemotherapy [18]. Bisphosphonate has been used for the treatment of clinical symptoms caused by bone metastasis and the complications. It suppressed the production of proliferative factors by bones through the suppression of the reabsorption of bone and thus it suppressed the proliferation of cancer cells but more studies seem necessary to confirm these data.

## Conclusion

The present case exhibits an unusual pathological behavior of gastric carcinoma: extensive bone metastasis in the absence of prior digestive symptoms. Metastasis to the bone is relatively rare with gastric carcinoma. However, in bone metastasis with an unknown source, detailed anamnesis and physical examination, hematologic and radiologic tests together with histopathologic investigation may be helpful for diagnosis. In conclusion, the prognosis of bone metastasis caused by gastric cancer is very poor and the prognosis of patients may be worsened due to a delayed diagnosis. Hence, tests to assess for bone metastasis are required for gastric cancer patients at the time of the initial diagnosis and the postsurgical follow-up observation and bone scintigraphy with its high sensitivity appears to be most useful. In such a manner, it is anticipated that the survival period as well as the quality of life may be improved with making a rapid diagnosis and by administering radiation therapy and other appropriate treatments. Studies with a large number of larger subjects are required to assess the effect of improving the survival period according to different treatment methods.

## Competing interests

The authors declare no competing interests.
